# Primary Care Skin Lesion Referrals Assessed via Teledermatology: A Retrospective Analysis

**DOI:** 10.7759/cureus.90849

**Published:** 2025-08-24

**Authors:** Iman Hamid, Isabelle Vandormael, Ausama Atwan, Caroline Mills

**Affiliations:** 1 Department of Dermatology, St Woolos Hospital, Newport, GBR

**Keywords:** basal cell carcinoma (bcc), discharge, melanoma, primary health care, routine referrals, seborrheic keratosis, squamous cell carcinoma (scc), teledrmatology, urgent referrals, urgent suspected cancer

## Abstract

Introduction

Teledermatology has become a vital component of dermatologic care in the United Kingdom, particularly following the COVID-19 pandemic. It offers an efficient, remote alternative to face-to-face consultations by utilising high-quality clinical and dermoscopic images to assess and triage skin lesions. This study evaluates the effectiveness of teledermatology referrals through both urgent suspected cancer (USC) and routine pathways, focusing on diagnostic accuracy, referral appropriateness, and patient outcomes within the dermatology service.

Methods

Between January and March 2022, 383 teledermatology referrals for suspected skin malignancies were reviewed. Of these, 194 (50.7%) were USC referrals and 189 (49.3%) were routine referrals. All referrals were reviewed and triaged by a consultant dermatologist based on the history provided and available clinical images. Patients were subsequently scheduled for professional photography, including dermoscopy images. Diagnoses and management plans were made following consultant review, with outcomes including discharge, minor surgery, referral to other specialities, or in-person consultation.

Results

A total of 47 different dermatological diagnoses were identified. Among USC referrals, only 16% were confirmed as high-risk skin cancers, including squamous cell carcinoma (8.8%), malignant melanoma (5.7%), and lentigo maligna (1.5%). In contrast, 5.8% of routine referrals were later identified as high-risk lesions, with 2.7% confirmed histologically as malignant. Notably, benign conditions such as seborrhoeic keratosis were frequently referred via both pathways. Basal cell carcinoma (BCC) was referred to as USC, despite guidelines recommending routine referral for most BCC cases. This suggests substantial overuse of the urgent pathway.

Outcomes showed that 83.7% of patients were managed without requiring face-to-face appointments. About 44.6% were discharged with reassurance, 20.9% underwent minor surgery, and 1.3% required referral to surgical specialities due to lesion complexity or location. Only 15.6% were seen in “see and treat” clinics.

Discussion

The data underscore a significant issue of inappropriate urgent referrals for benign skin conditions, contributing to delays and resource strain in secondary care. Contributing factors include limited dermatology training in primary care, fear of misdiagnosis, and patient pressure. Enhancing GP education, developing standardised referral guidelines, and improving access to dermoscopic imaging tools in primary care could help mitigate these challenges. Teledermatology not only enhances diagnostic accuracy but also offers cost savings, with previous local studies estimating a reduction of £43 per patient.

Conclusion

The rising incidence of skin cancer, coupled with limited dermatology training for primary care providers, fear of misdiagnosis, and patient anxiety, strains dermatology services. Our study demonstrates that appropriate referrals to teledermoscopy service will help optimise outpatient resources and facilitate prompt treatment for patients with suspected high-risk skin cancers.

## Introduction

Teledermatology has seen widespread adoption across dermatology departments in the United Kingdom, particularly following the COVID-19 pandemic. It has enabled continuity of care while minimising in-person contact, helping services maintain pre-pandemic levels of care. The core aim of teledermatology is to provide timely patient assessment, reduce unnecessary hospital visits, and ease pressure on outpatient clinics by using dermoscopic imaging for remote diagnosis and effective triage. This approach helps prioritise face-to-face consultations for patients who need them most.

Numerous studies have demonstrated teledermatology’s effectiveness in triaging patients and reducing waiting lists, thereby improving access to secondary care and enhancing the efficiency of healthcare delivery. The inclusion of dermoscopic images in teleconsultations has been shown to improve diagnostic accuracy significantly. In a study by Ferrándiz et al., appropriate management decisions were made in 94.3% of cases when dermoscopic images were included, compared to 79.2% without them [1].

In primary care, general practitioners (GPs) typically refer patients with skin lesions either via the urgent suspected cancer (USC) (two-week wait (2WW)) pathway for suspected malignant melanoma (MM) and squamous cell carcinoma (SCC) lesions or as routine referrals for less concerning lesions. A cross-sectional study by Le Roux et al. found that 14.2% of GP consultations involved skin-related issues, with common presentations including both benign and malignant skin lesions, infections, and eczema [2]. This trend is reflected in dermatology, where workloads are increasing in parallel with the rising incidence of skin cancer. Skin cancer alone now accounts for approximately 50% of the workload in secondary care dermatology [3].

The primary objective of this study was to compare outcomes of teledermatology referrals referred through USC versus the routine pathway, to identify the most frequent skin conditions seen, and to determine whether the referrals were appropriately conducted through the correct pathway. The secondary objective involves reviewing the rate of high-risk cancers inappropriately referred through the routine pathway.

## Materials and methods

This retrospective study examines a snapshot of teledermatology referrals to St Woolos Hospital from various general practices across the Newport area in Wales. Between January and March 2022, we reviewed 383 skin lesion referrals submitted using the local pathway for teledermoscopy service. The inclusion criteria include patients referred to teledermatology as suspected skin cancer lesions, either through the routine or USC pathway. Of these, 189 cases (49.3%) were referred as routine (for non-melanoma/non-SCC lesions), while 194 cases (50.7%) were classified as urgent or USC for MM lesions or SCC. Collected data included patient demographics, age, referral type, provisional diagnosis and the outcome/management recommended.

In accordance with the referral pathway, all submissions were initially triaged by a consultant dermatologist and either upgraded or downgraded based on the clinical history provided. In a minority of cases, standard clinical photographs were included by the referring GP to support the assessment. Referrals that did not meet the criteria for the 2WW pathway were downgraded to routine. Following triage, patients were scheduled for a medical photography appointment based on the urgency of their referral. The average waiting time was approximately two weeks for urgent cases and between six and 12 months for routine referrals. These patients were then reviewed at one of our designated medical photography hub clinics, where high-resolution clinical and dermoscopic images were taken and uploaded to their electronic medical records.

Following professional photography, each case was reviewed by a consultant dermatologist within a few days in both cohorts to determine a potential diagnosis and appropriate management. The outcome of the teleconsultation was communicated by a letter to both the patient and their GP. Based on the consultant dermatologist's assessment of the teledermoscopy images, patients were either discharged, scheduled for a minor surgical procedure, referred to another speciality, or booked for a face-to-face consultation.

## Results

**Figure 1:**
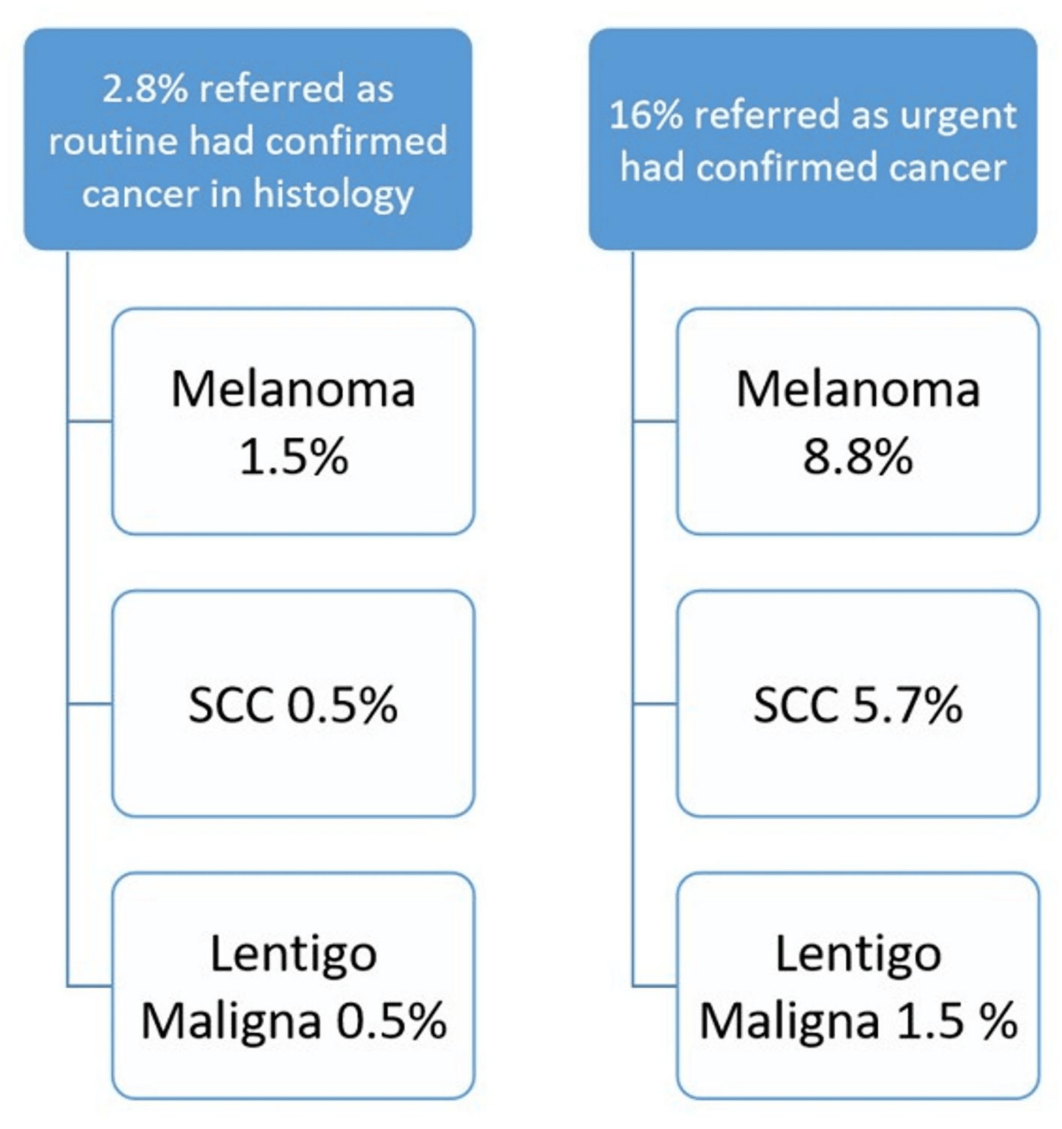
Urgent versus routine referrals, high-risk cancer outcomes

A total of 47 different diagnoses were identified across both cohorts. Among patients referred via the urgent or USC pathway, only 16% were found to have high-risk skin cancers, comprising 8.8% SCC, 5.7% MM, and 1.5% lentigo maligna (LM). The remaining USC referrals were diagnosed with benign lesions or non-urgent skin conditions (see Figure 1).

In comparison, 5.8% of patients referred through the routine pathway were suspected to have high-risk skin cancers after teledermoscopy review of their professional photographs. These individuals were offered an urgent face-to-face appointment through one of our "see and treat" clinics, where patients are informed that they may undergo a procedure during the same visit. Of this group, 2.7% were confirmed histologically to have high-risk malignancies, including MM (1.5%), SCC (0.5%), and LM (0.5%).

On average, patients referred through the routine pathway experienced delays of more than six months before being seen for professional clinical and dermoscopic imaging, followed by consultant review a few days later. These delays are largely attributed to the increasing demand on secondary care services, especially after the COVID-19 pandemic and also to the extensive geographical area covered by our dermatology service.

Seborrhoeic keratosis, a benign condition that does not typically require secondary care referral, was also referred through both pathways, 20.6% via urgent/USC and 21.2% via the routine pathway, which is surprisingly a very similar percentage in both pathways. Similar results were observed in a study by Elaribi et al. [4]. 

In addition to variant benign skin lesions and benign moles, a few inflammatory conditions were incorrectly referred to as suspected skin cancer through the skin lesion referral pathway (Table 1).

**Table 1:** Different diagnoses reviewed in routine versus urgent/urgent suspected cancer (USC) referrals

Diagnosis	Routine n (%)	Urgent/USC n (%)
Basal cell carcinoma (BCC)	46 (24.4)	46 (23.7)
Seborrhoeic keratosis	40 (21.2)	40 (20.6)
Actinic keratosis	12 (6.3)	1(0.5)
Malignant melanoma	8 (4.2)	11 (5.7)
Squamous cell carcinoma (SCC)	3 (1.5)	17 (8.8)
Benign naevus	13 (6.9)	18 (9.3)
Lentigo maligna	1 (0.5)	3 (1.5)
Dermatofibroma	7 (3.7)	7 (3.6)
Lichenoid keratosis	4 (2.1)	5 (2.6)
SCC in situ	5 (2.6)	2 (1.0)
Atypical naevus	9 (4.7)	8 (4.1)
Melanonychia	2 (1.0)	1 (0.5)
Angioma	1 (0.5)	3 (1.5)
Lentigines	3 (1.5)	1 (0.5)
Other benign lesion	1 (0.5)	4 (2.1)
Haematoma	0	4 (2.1)
Viral wart	1 (0.5)	3 (1.5)
Sebaceous hyperplasia	3 (1.5)	0
Giant comedo	1 (0.5)	1 (0.5)
Chondrodermatitis	1(0.5)	0
Scar	1 (0.5)	1 (0.5)
Myxoid cyst	1 (0.5)	0
Spitz naevus	0	1 (0.5)
Fibrous papule	2 (1.0)	0
lymphangioma	0	2 (1.0)
Clear cell acanthoma	0	1 (0.5)
Inflammatory conditions	6 (6.2)	2 (1.0)
Cutaneous horn	0	1 (0.5)
Foot lesion	1 (0.5)	0
Others	18 (9.5)	11 (5.6)

**Figure 2:**
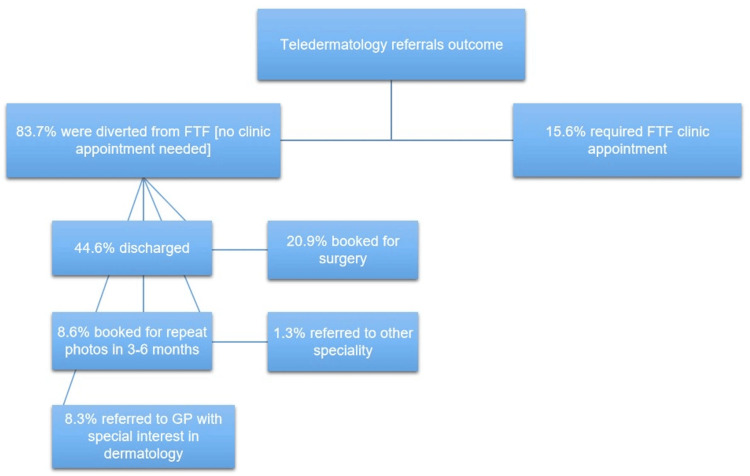
Outcome of overall teledermatology referrals

Overall, 83.7% of all the referrals were diverted from having a face-to-face appointment, opening up more clinic slots for new referrals; 44.6% of them were discharged with reassurance letters with no need for further face-to-face appointment; while only 15.6% were seen in face-to-face which have a more diagnostic accuracy [5]; 20.9% were directly booked for minor surgery at the dermatology department, waiting time for surgery depends on the level of suspicion of the lesion; 8.3% were referred to local enhanced services (LES) led by GPs with a special interest in dermatology for low-risk procedures on trunk and limbs; 1.3% were referred to other surgical specialties (plastics surgery, maxillofacial, and oculoplastics) due to the size of the lesion or it’s anatomical location; and 4.1% of patients were booked for teledermoscopy digital monitoring with repeated photographs three to six months later (Figure 2). 

## Discussion

As per NICE guidelines, MMs and SCCs should be referred to the USC pathway. The majority of USC referrals in this study were identified as benign or non-urgent skin lesions 81.3%. Similar findings have been found in a study by Elliot et al., where 87% of the 2WW referrals were considered non-MM/SCC or other malignancy, and only 13% were highly suspected cancer lesions [6]. 

Our study highlights the importance of adequate dermatology training for primary care clinicians, which could play a significant role in reducing waiting lists. Accurate patient selection for the appropriate teledermoscopy referral pathway (routine versus USC) can minimise unnecessary referrals for low-complexity benign lesions such as seborrhoeic keratoses and vascular lesions. This, in turn, helps prioritise limited face-to-face dermatology appointments for high-risk cases [7], ultimately reducing waiting times for face-to-face consultations [8].

Possible explanations for these inappropriate skin lesion referrals are inadequate dermatology training at the primary care level, anxiety of misdiagnosing a malignant lesion and pressure from anxious patients. This, in turn, leads to an increased demand for dermatology services with increasing waiting times, especially for routine referrals.

The fact that primary care clinicians also receive outcome letters for patients' teledermatology consultations is a valuable opportunity to receive feedback and develop their clinical diagnostic skills for various skin lesions. The level of satisfaction and comfort with the teledermatology experience was not only high among patients [9,10], but it was also high among clinicians. The service received positive feedback from referring GPs [11] as teledermatology provided an opportunity for less experienced GPs to enhance their diagnostic skills [12].

Our Dermatology Department was among the first in the UK to adopt teledermatology services. For over 10 years, we have successfully utilised high-quality clinical and dermoscopic images to triage and manage patients, demonstrating both clinical effectiveness and cost-efficiency. A study conducted in our department in 2019 by Lowe et.al showed that reviewing patients via teledermatology service resulted in savings of £43 per patient, leading to a total net saving of £170,280 for that year [13]. Teledermatology cost-efficiency was also reviewed in other studies [14]. We believe that referring patients through appropriate pathways and avoiding unnecessary referrals for benign conditions such as seborrhoeic keratosis can lead to even greater savings. Overall, teledermatology has had a positive impact on both patient care and NHS services [15].

Some of the limitations of this study include the variability in GP diagnostic skills, which can affect the quality of teledermatology referrals, as differences in experience and training influence clinical judgment; this was not included in the study. Additionally, the generalisability of the results is also constrained, as the study setting may not reflect other healthcare environments with differing resources, patient populations, or access to technology. These factors highlight the need for further research to evaluate standardised GP training and implementation in diverse settings.

## Conclusions

To help reduce the pressure on secondary care and waiting time to teledermatology service further, we recommend proper training for GPs in diagnosing common skin lesions, with an emphasis on the most common benign lesions like seborrhoeic keratoses, developing guidelines to help them determine the urgency of referrals, creating a standardise proforma to help guide clinicians in providing comprehensive history and clinical information about the lesion and lastly to include clinical photos with referrals can aid dermatologists at secondary care in effectively triaging referrals. However, if GPs have significant concerns, they can still refer patients directly to the 2WW pathway.

Additionally, arranging for high-quality clinical and dermoscopic images to be taken at the primary care level, for example, by providing GPs with a mobile phone adaptor and dermatoscope to capture images of the lesion in question, is most likely to play a key role in reducing waiting times for patients referred in both cohorts, especially those referred as routine.
